# Assessing mechanical catheter dysfunction in automated tidal peritoneal dialysis using cycler software: a case control, proof-of-concept study

**DOI:** 10.1038/s41598-022-09462-9

**Published:** 2022-04-05

**Authors:** Krystell Oviedo Flores, Lukas Kaltenegger, Fabian Eibensteiner, Markus Unterwurzacher, Klaus Kratochwill, Christoph Aufricht, Franz König, Andreas Vychytil

**Affiliations:** 1 Division of Nephrology and Dialysis, Department of Medicine III, Medical University of Vienna, Währinger Gürtel 18-20, 1090 Vienna, Austria; 2 Baxter Healthcare GmbH, Vienna, Austria; 3 Division of Pediatric Nephrology and Gastroenterology, Department of Pediatrics and Adolescent Medicine, Comprehensive Center for Pediatrics, Medical University of Vienna, Vienna, Austria; 4 Christian Doppler Laboratory for Molecular Stress Research in Peritoneal Dialysis, Medical University of Vienna, Vienna, Austria; 5 Center for Medical Statistics, Informatics and Intelligent Systems, Medical University of Vienna, Vienna, Austria

**Keywords:** Health care, Medical research, Nephrology

## Abstract

New recommendations on evaluation of peritoneal membrane function suggest ruling out catheter dysfunction when evaluating patients with low ultrafiltration capacity. We introduce the use of a combination of parameters obtained from the cycler software PD Link with HomeChoicePro (Baxter International Inc., Illinois, United States) cyclers for predicting catheter dysfunction in automated peritoneal dialysis patients (APD). Out of 117 patients treated at the Medical University of Vienna between 2015 and 2021, we retrospectively identified all patients with verified catheter dysfunction (n = 14) and compared them to controls without clinical evidence of mechanical catheter problems and a recent X-ray confirming PD catheter tip in the rectovesical/rectouterine space (n = 19). All patients had a coiled single-cuff PD catheter, performed tidal PD, and received neutral pH bicarbonate/lactate-buffered PD fluids with low-glucose degradation products on APD. Icodextrin-containing PD fluids were used for daytime dwells. We retrieved cycler data for seven days each and tested parameters' predictive capability of catheter dysfunction. Total number of alarms/week > 7 as single predictive parameter of catheter dislocation identified 85.7% (sensitivity) of patients with dislocated catheter, whereas 31.6% (1-specificity) of control patients were false positive. A combination of parameters (number of alarms/week > 7, total drain time > 22 min, ultrafiltration of last fill < 150 mL) where at least two of three parameters appeared identified the same proportion of patients with catheter dislocation, but was more accurate in identifying controls (21.1% false positive). In contrast to yearly PET measurements, an easily applicable combination of daily cycler readout parameters, also available in new APD systems connected to remote monitoring platforms shows potential for diagnosis of catheter dysfunction during routine follow-up.

## Introduction

Successful treatment and technique survival on peritoneal dialysis (PD) depend on a long-term peritoneal access that provides effective peritoneal drainage of dialysis fluid for maintenance of an adequate fluid and salt homeostasis^1^. PD catheter tip migration is a major complication that usually requires catheter reposition or replacement and may eventually lead to technique failure^2^. Catheter dislocation may be associated with slow and incomplete drainage of dialysis fluid from the peritoneal cavity. It is important to note that while drain volume can be larger than fill volume even with malpositioned catheters^3^, drainage may be incomplete, leading to increased abdominal pressure and a higher risk of hernias and dialysate leaks^4^.

Low ultrafiltration (UF) capacity may be misleading in patients with PD catheter dysfunction as incomplete peritoneal drainage of dialysate can result in insufficient ultrafiltration and fluid overload. Therefore, the recent ISPD recommendations suggest that low UF capacity should be evaluated by considering mechanical drainage problems first, before suspecting membrane dysfunction^5^. Previous publications have suggested the association of slow drainage of PD fluid, poor net UF or constant alarms during cycler treatment with catheter dysfunction^6,7^. However, until now no standard procedure has been systematically evaluated on the usability to assess mechanical drainage problems in PD patients.

In contrast to isolated measurements of ultrafiltration capacity, for example during the peritoneal equilibration test (PET), automated peritoneal dialysis (APD) cycler readouts allow meticulous daily analysis of important treatment parameters. The aim of this proof-of-concept study was to analyze the usability of cycler parameters routinely used in our center for identifying APD patients with catheter malfunction.

## Results

Out of 117 APD patients treated between 2015 and 2021, 14 patients with verified catheter dysfunction (12%) and 19 controls (16%) could be identified. There were no significant differences in demographic data (Table 1) between both groups. The median daily glucose load was 132.0 g/day [IQR (73.6)] in the controls, and 124.1 g/day [IQR (8.0)] in the cases (MWU test, *P* = 0.0471). Other PD prescription data were not statistically different between groups (Table 2). Eight out of the 14 patients with catheter dysfunction could continue PD treatment after conservative measures (reduction of tidal volume, laxatives and exercises in order to relocate catheter tip). Four patients needed catheter repositioning or replacement (laparoscopically) and 2 patients were transferred to hemodialysis since it was not possible to continue with PD.

**Table 1:** Demographic data and biochemical parameters at the start of the observation period.

Characteristics	Cases (*n* = 14)		Controls (*n* = 19)	
Age (years), mean (SD)	57.07	(13.42)	49.37	(15.80)
**Gender, *n* (%)**				
Female	4	(28.57)	7	(36.84)
Male	10	(71.43)	12	(63.16)
**Cause of kidney disease, *n* (%)**				
Glomerulonephritis	6	(42.86)	5	(26.32)
Polycystic kidney disease	1	(7.14)	4	(21.05)
Vascular nephropathy	2	(14.29)	1	(5.26)
Diabetic nephropathy	2	(14.29)	4	(21.05)
Glomerular amyloidosis	0	(0.00)	2	(10.53)
Other/unknown	3	(21.43)	3	(15.79)
Comorbidities *n* (%)				
Cardiovascular history	5	(35.71)	9	(47.37)
Hypertension	10	(71.43)	12	(63.16)
Diabetes	4	(28.57)	6	(31.58)
Time on PD (months), median (IQR)	3.0	(8.89)	3.0	(0.00)
**Biochemical parameters (mean, SD)**				
D/P Creatinine	0.81	(0.09)	0.80	(0.12)
GFR^a^ (ml/min/1.73 m^2^)	4.60	(1.90)	4.30	(2.50)
Weekly Kt/V	2.03	(0.29)	2.28	(0.45)
Weekly CCr (L/week/1.73 m^2^)	72.39	(15.69)	78.74	(26.20)
Serum creatinine (mg/dL)	7.93	(2.40)	7.67	(2.65)
BUN (mg/dL)	52.87	(17.37)	49.96	(15.66)
Serum calcium (mmol/L)	2.16	(0.13)	2.22	(0.13)
Serum phosphate (mmol/L)	1.89	(0.60)	1.65	(0.57)
Albumin (g/L)	34.76	(4.31)	33.55	(6.02)
Hemoglobin (g/dL)	10.18	(1.09)	10.62	(1.35)
WBC (G/L)	6.54	(1.37)	6.13	(1.67)

^a^The mean GFR was calculated as average value of creatinine and urea clearance using 24-h urine collections. For all parameters, *P* values > 0.05 when comparing cases vs controls (two-tailed MWU test). BUN, blood urea nitrogen; CCr, creatinine clearance; GFR, glomerular filtration rate; WBC, white blood cell counts.

**Table 2:** APD treatment parameters.

Parameter	Cases (*n* = 14)		Controls (*n* = 19)	
Last fill volume with icodextrin (mL), median (IQR)	1000	(125)	1000	(500)
Treatment volume ^a^ (mL), median (IQR)	9500	(0)	9500	(4500)
Fill volume (mL), median (IQR)	2000	(315)	1997	(501)
Drain volume (mL), median (IQR)	2080	(384)	2065	(1062)
Tidal volume (%),median (IQR)	57.5	(30.0)	60.0	(20.0)
Tidal volume (mL), median (IQR)	1090	(525)	1200	(600)
Daily glucose load ^b^ (g/day), median (IQR)	124.1	(8.0)	132.0	(73.6)

All patients performed tidal PD and received icodextrin-containing PD fluid for the daytime dwell, except for one patient in the control group who performed nightly intermittent PD without last fill. For all parameters, *P* values > 0.05 when comparing cases vs controls (two-tailed MWU test), except for daily glucose load (*P* = 0.0471).

^a^Last fill with icodextrin excluded.

^b^Daily glucose load was calculated as the total glucose in the fresh PD fluid infused each day in grams.

### Number of alarms

The mean number of cycler alarms was 19.3 ± 16.5/week in the cases and 5.2 ± 4.0/week in the controls, with a median of 13.5 alarms/week [IQR (16.8)] and 5.0 alarms/week [IQR (6.0)], MWU test *P* = 0.0002).

### Total drain time and drain volume at the end of the cycler session

Total drain volumes at the end of cycler treatment were not statistically different between groups, with a median of 2080 mL [IQR (384)] in the cases and 2065 mL [IQR (1062)] in the controls (MWU test *P* = 0.7329). However, the total drain time at the end of the cycler session was significantly larger in the cases than in the controls (mean 29.0 ± 11.3 min vs 20.2 ± 6.8 min, *P* = 0.0012) and a median of 24.3 min [IQR (14.3)] vs 17.7 min [IQR (5.3)].

### Net UF of last fill (daytime dwell)

All patients received icodextrin-containing PD fluid for the daytime dwell, except for one patient in the control group who performed nightly intermittent PD without last fill. The mean net UF of the cases was statistically different and more negative than in the controls (− 12.7 ± 153.3 mL vs 206.3 ± 228.4 mL, *P* = 0.0047). The median net UF of last fill was − 10.0 mL [IQR (259.9)] in the cases vs 227.6 mL [IQR (328.9)] in the controls.

### Number of days with negative UF of last fill per week

The number of days with negative UF of last fill (drain volume lower than fill volume) tended to be higher in the cases than in the controls (mean 3.3 ± 2.6 days/week vs 1.6 ± 2.1 days, MWU test *P* = 0.0749). The median for the cases was 3.0 days/week [IQR (6.0)] vs 1.0 day/week [IQR (2.5)], respectively.

### Net UF and gcUF during cycler treatment (without UF of last fill)

The mean net UF per cycler treatment in controls was 393.5 ± 445.8 mL, whereas the cases had a lower mean net UF of 102.2 ± 372.2 mL per treatment (median 242.1 mL [IQR (607.6)] vs 160.5 mL [IQR (826.1)], *P* = 0.1320). Nevertheless, the mean glucose-corrected UF (gcUF) was not statistically different between groups, 1.5 ± 2.9 mL/g/day in the controls vs. 0.06 ± 2.3 mL/g/day in the cases (median 1 mL/g/day [IQR (4.3)] vs 0.42 mL/g/day [IQR (3.3)], *P* = 0.2258).

Supplementary Table S1 provides a summary of all analyzed parameters.

### Defining cut-off values for prediction

The parameters with the highest area under the curve (AUC) were number of total alarms (0.87, *P* = 0.0004), total drain time (0.82, *P* = 0.0017), and mean net UF of last fill (0.79, *P* = 0.0056). We decided to include number of days with negative UF of last fill (0.68, *P* = 0.0839) because there was a statistical trend towards significance. Furthermore, in our experience, it is easy to report in clinical practice for both, patients and physicians. The smallest AUC was identified with the mean gcUF (0.63; *P* = 0.2155). Therefore, this parameter was not included in further analysis.

**Figure 1:**
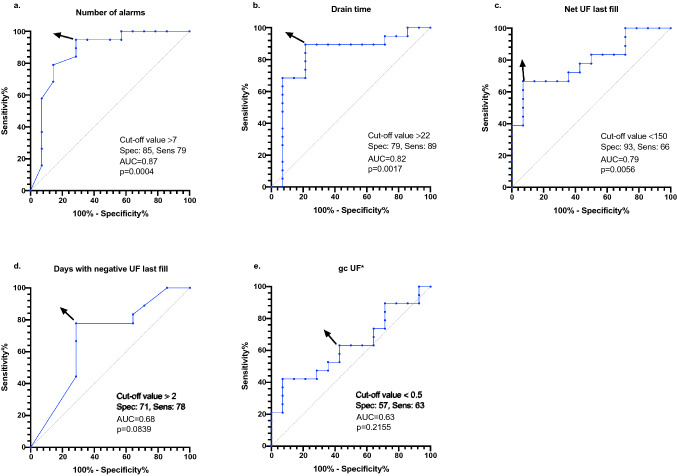
Receiver operator characteristic (ROC) curves for the parameters obtained from APD cycler card management software in the cases and the controls. The arrow indicates the point corresponding to the cut-off value selected. AUC = area under the curve. (**a**) Total number of alarms per week; (**b**) total drain time at the end of cycler session; (**c**) mean net UF of last fill; (**d**) days with negative UF of last fill; (**e**) mean gcUF during cycler treatment (*not included in further analysis).

For these four selected parameters the cut-off values for predicting catheter dislocation based on ROC curves were > 7 alarms/week (specificity: 85%, sensitivity: 79%), > 22 min drain time at end of the cycler session (specificity: 79%, sensitivity: 89%), < 150 mL mean net UF of last fill (specificity: 93%, sensitivity: 66%), and > 2 days per week with negative UF of last fill (specificity: 71%, sensitivity: 78%) (Fig. 1, Supplementary Table S2).

Table 3 summarizes the simple and stepwise logistic regression of the parameters for predicting catheter dislocation. Each additional alarm per week increased the odds of catheter dislocation by a factor of 1.298 (95% CI 1.062; 1.586, *P* = 0.011) leading to an increase by 40.6 for 14 alarms (mean difference between cases and controls). Each additional minute of total drain time increased the odds of catheter dislocation by a factor of 1.125 (95% CI 1.015; 1.247, *P* = 0.025). A 1 ml (ml) decrease of mean UF of last fill increased the odds for catheter dislocation by a factor of 1.006 (95% CI 0.989; 0.999, *P* = 0.013). The mean difference between cases and controls was approximately 200 mL leading to an increase of the odds by 3.33 for a catheter dislocation. Furthermore, an increase of days with negative UF of last fill tended to be associated with catheter dislocation (*P* = 0.063). In the stepwise logistic regression analysis (including only the four parameters) the total number of alarms improved the fit of the model for the prediction of catheter dislocation compared to the null model. DeLong test showed a significant difference between the two AUCs (P<0.001).

**Table 3:** Summary of simple and stepwise logistic regression of the parameters for predicting catheter dislocation.

Parameter	Simple logistic regression			Stepwise logistic regression		
	AUC [95% CI]	OR [95% CI]	*P* value	ß-coefficient	OR [95% CI]	*P* value
Total alarms	0.87 [0.73–1.00]	1.298 [1.062; 1.586]	0.011	0.261	1.298 [1.062; 1.586]	0.011
Total drain time (min)	0.82 [0.66–0.99]	1.125 [1.015; 1.247]	0.025	0.117		NC
Net UF last fill (mL)^a^	0.79 [0.63–0.95]	0.994 [0.989; 0.999]	0.013	-0.006		NC
Days with negative UF last fill	0.68 [0.48–0.88]	1.345 [0.984; 1.839]	0.063	0.296		NC

^a^Corresponding to daytime dwell. AUC, area under the curve; CI, confidence interval; NC, no candidate; OR, odds ratio; SE, standard error.

After adding the standard deviation of the residuals (SD-Res) of the net UF of the last fill as well as the SD-Res of the gcUF as measures of variability of the net UF to the existing four covariates into the stepwise regression model and using BIC as criterion for selection, all covariates except total alarms were eliminated, indicating that SD-Res does not improve the prediction model.

One patient with catheter dysfunction developed a peritonitis during the second part of the study period. We repeated analysis of the ROC curves and stepwise logistic regression analysis after excluding this patient, and the results were consistent.

### Predictive power for combination of parameters

To facilitate the utilization of parameters of the model in clinical practice, models with the highest AUCs were selected for parameter combinations: total alarms/week, drain time at the end of the cycler session, UF of last fill (or, alternatively, days with negative UF of last fill). Total number of alarms/week > 7 as single predictive parameter identified 85.7% of the cases, 31.6% of controls were false positive. The presence of at least two of three parameters (number of alarms/week > 7, total drain time > 22 min, UF last fill < 150 mL) identified the same proportion of cases compared with number of alarms alone (85.7%), but it was more accurate in identifying control patients (21.1% vs 31.6% false positive). Stricter definitions, such as fulfilling all three criteria, reduced the predictive power for diagnosing catheter dislocation, but also decreased the number of false positive controls (Table 4).

**Table 4:** Combination of the most promising parameters  for identifying patients with catheter dysfunction in the cases and the controls.

	Only alarms as criterion fulfilled	At least 1 of 3 criteria fulfilled	At least 2 of 3 criteria fulfilled	3 of 3 criteria fulfilled
**Combination 1:** Number of alarms > 7, Drain time > 22 min, UF last fill < 150 mL				
Cases correctly identified	85.7%	100%	85.7%	71.4%
False positive controls	31.6%	57.9%	21.1%	5.3%
**Combination 2:** Number of alarms > 7, Drain time > 22 min, Days with negative UF last fill > 2				
Cases correctly identified	85.7%	100%	78.6%	57.1%
False positive controls	31.6%	52.6%	15.8%	5.3%

## Discussion

We demonstrated that a protocol assessing several cycler readout parameters might be a feasible, easy-to-perform tool to adequately screen APD patients for catheter dysfunction.

In this proof-of-concept study, we confirmed the clinically observed impression that the parameters under investigation serve as predictors for diagnosis of catheter dysfunction. This is the first controlled analysis evaluating parameters obtained from the APD cycler software for the diagnosis of catheter dysfunction in two well-defined patient groups. The selected cases and controls represent typical APD patients with radiological confirmation of PD catheter position and clinical assessment of catheter performance.

Patients with a malfunctioning catheter may clinically present with slow drainage of PD fluid, prolonged drain times, poor net UF^6,7^ or constant alarms during cycler treatment^8^, resulting in impairment of quality of life. Early diagnosis prevents the possibility of volume overload and complications associated with increased intraperitoneal volume that subsequently may lead to technique failure^9^.

Recent ISPD recommendations suggest that mechanical problems should be excluded prior to membrane function testing in patients with insufficient UF capacity. However, there exists no standard protocol on how to diagnose catheter dysfunction^5^.

One of the requirements for good catheter performance is an adequate position of the PD catheter tip. The ISPD guidelines on PD access recommend using the upper border of the pubic symphysis as a reference point for the ideal location of the catheter tip deep in the pelvic area^7^. During or after catheter implantation, the position of the catheter tip might be confirmed with a plain abdominal radiological examination, but this is not routinely performed in all centers^10^.

Single diagnostic tests performed in yearly intervals (e.g. ultrafiltration capacity during the PET) may not be suitable for diagnosing mechanical problems. Net ultrafiltration is more variable than peritoneal small solute transport. Availability of daily treatment data extracted from the APD cycler card software allows a better clinical evaluation of mechanical drainage problems in routine practice.

In our study, in the stepwise logistic regression analysis, only number of alarms/week was a statistically significant predictor of catheter dislocation. As the Pearson and Spearman correlation coefficients between number of total alarms and the other metric variables revealed values above 0.5 (indicating moderate to strong correlations), none of the other variables could contribute anything in addition. However, considering the limited patient number this analysis does not refute the importance of other parameters for diagnosing mechanical drainage problems.

A combination of at least two of three parameters which had significant ROC curves and an AUC of > 0.75 (number of alarms, total drain time, net ultrafiltration of last fill) could identify a similar number of patients with catheter dislocation compared to using the number of alarms alone (85.7%). However, when using a combination of parameters, the percentage of false positive control decreased (Table 4).

The results of this study are strengthened by the accordance of the cut-off values derived from the ROC curves with conclusions of other authors in the recent literature. We determined that a cut-off value of > 7 alarms/week is a good predictor of catheter dysfunction. According to Neri et al.^11^, the use of tidal PD optimized catheter flow function, resulting in the presence of < 1 alarm per day (corresponding to < 7 alarms per week). Other authors also suggest that outflow failure prompts alarms in APD patients treated with HomeChoicePro^8^. Moreover, the cut-off value for mean drain time of > 22 min for predicting catheter dysfunction found in our study is similar to the conclusion of other authors who suggested that a drainage time of less than 20 min is a marker of good catheter performance^3,12^. A sub-optimal catheter position results in poor hydraulic function that may cause outflow failure and can prolong drain time at the end of the cycler session^10,13^. We demonstrated that low or negative UF of last fill during the daytime dwell was evident in patients with catheter dislocation. Our selected cut-off value of UF for predicting catheter dysfunction of 150 mL per daytime dwell is consistent with data reported by Plum et al.^14^ who found a mean ultrafiltration volume of last fill of 278 ± 43 mL/day in APD patients treated with icodextrin during the long dwell, which was comparable with the mean net UF of last fill of our control group (206.3 ± 228.4 mL).

Our study has certain limitations. We acknowledge that we are reporting a retrospective, single-center study of patients on APD, which may limit statistical power and generalizability of our results. For example, albeit not statistically significant, assessing the number of treatment days with negative UF of last fill may be more practicable than analyzing cut-off values of ultrafiltration with icodextrin. Although we included all APD patients treated in our center between 2015 and 2021 who were suitable for inclusion in either groups, the sample size is still small and exclusion of 29% of eligible patients with missing data may have limited the validity of the stepwise logistic regression analysis and the representativeness of the results. Larger, prospective studies are required to confirm our clinical observations.

In conclusion, a timely intervention in patients with catheter dysfunction may prevent adverse events with potential negative impact on technique survival^9^. In this context, the use of total number of alarms/week as well as a combination of parameters derived from the APD cycler card management software with the selected cut-off values are good predictors for assessing catheter function. Our findings may be especially interesting because these parameters are also available in new APD cyclers with devices connected to remote monitoring platforms, which are rapidly gaining importance and availability^9,15^.

## Material and methods

### Study design

This retrospective case–control, proof-of-concept study included patients with end-stage chronic kidney disease treated with HomeChoicePro APD system (Baxter International Inc., Illinois, United States) at the Division of Nephrology and Dialysis, Medical University of Vienna.

To validate parameters related to mechanical drainage problems we studied two well-characterized patient groups using data retrieved from the APD cycler card management software PD Link (Baxter International Inc., Illinois, United States). In order to minimize bias, retrospective analysis of the PD catheter position based on posteroanterior and/or lateral abdomen X-ray, if available, and review of clinical records to assess catheter drainage function and patients’ demographic data was performed by two investigators. This study was performed in line with the principles of the Declaration of Helsinki and involves retrospectively collected data. Informed consents were collected from all our patients before inclusion in our database and biobank. Approval was granted by the Local Ethics Committee of the Medical University of Vienna (study protocol EK 2035/2015).

### Patient selection

**Figure 2:**
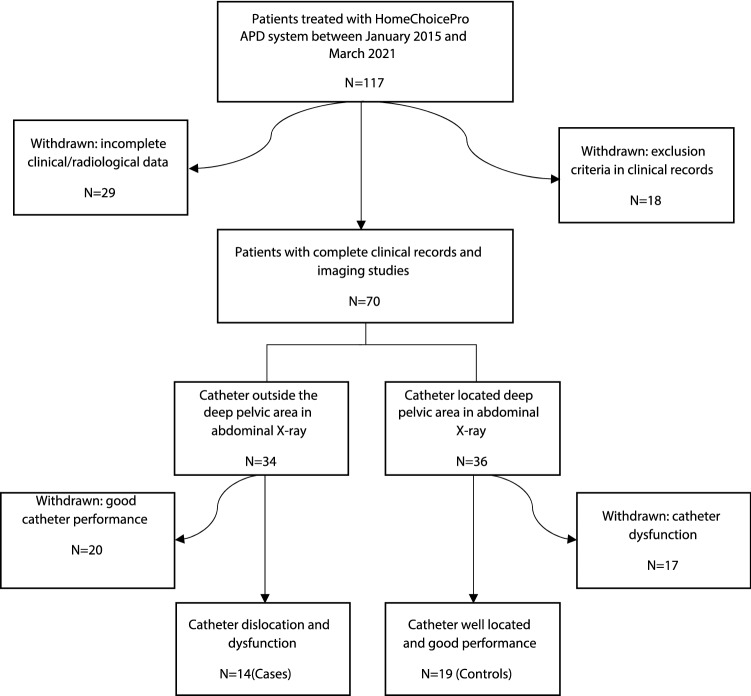
Flow chart of cases and controls selection.

Patients included in this study received regular APD between January 2015 and March 2021 at the Medical University of Vienna. All patients were treated with neutral pH bicarbonate/lactate-buffered PD solutions with low concentration of glucose degradation products (GDP) (Physioneal 40, Baxter Healthcare) and icodextrin-containing PD fluids (Extraneal, Baxter Healthcare) for the daytime dwell (except for one patient in the control group who performed nightly intermittent PD without last fill). All patients had a coiled single-cuff PD catheter and performed tidal peritoneal dialysis. Patients with incomplete data (no abdominal X-ray or data on catheter performance available) were withdrawn. Patients with a history of large open abdominal or pelvic surgery, ascites, malignant pelvic neoplasia or death within 15 days after APD start, were excluded (Fig. 2).

### Cases

All patients with clinical evidence of drainage problems that required a diagnostic and/or therapeutic intervention (repeated abdominal X-ray to verify catheter position, use of laxatives, catheter repositioning or replacement) and with confirmed catheter dislocation in an abdominal X-ray (verifying catheter tip dislocation outside the deep pelvic area) were selected as cases.

### Controls

Patients with an uncomplicated course of treatment without clinical evidence of mechanical problems and a recent routine X-ray confirming position of the PD catheter tip in the recto-vesical/ recto-uterine space were selected as controls. Patients with a computed tomography (CT) peritoneography or an explorative laparoscopy/adhesiolysis after start of PD were excluded from the control group since requirement of these diagnostic or therapeutic measures do not represent an uncomplicated course of treatment.

### Parameters

Based on previous publications^8,9,11,15,16^ and on our own clinical expertise, daily cycler readout parameters are routinely used at our center to detect mechanical drainage problems early during routine follow-up. Number of alarms, drain time at the end of cycler session, net UF from last fill, number of days with negative UF of last fill and net UF from cycler treatment (net UF and glucose-corrected UF [gcUF]) were analyzed in the present study.

The observation period for both groups consisted of seven days of continuous APD treatment. Among cases, these parameters were analyzed in the week immediately before the diagnostic imaging studies confirming catheter tip migration. The time-period for the analysis of the control group was seven days selected randomly depending on data availability, within a pre-defined time window between 1 and 3 months after the start of PD. This time window was chosen considering the appearance of changes of peritoneal membrane function parameters early after PD start that may influence ultrafiltration^5^.

Furthermore, daily glucose load was calculated as total grams of glucose in the fresh PD fluid infused each day. Daily glucose corrected ultrafiltration (gcUF) was calculated as UF in mL divided by glucose load in grams. APD prescription was not modified during the observation period.

### Statistical analysis

Statistical analysis was performed using IBM SPSS Statistics (IBM Corp., Armonk, NY, USA), R (GNU General Public License^17^) and GraphPad Software (California, USA). Results were expressed as relative frequencies for categorical variables, mean with standard deviation (SD) for continuous variables, and median with interquartile range (IQR) for skewed distributions. Comparison of categorical variables was performed with Fisher’s exact test. Continuous variables were analyzed with Mann–Whitney U-test (MWU). The receiver-operating curves (ROC) for each parameter were computed from the observed proportion utilizing Clopper method^18^, without correction for multiple comparisons of area calculation. Selected cut-off values for the parameters were identified via means of the highest sensitivity and specificity. Stepwise logistic regression (catheter dislocation versus control as dependent variable) was performed to evaluate the contribution of several covariates to anticipation of catheter dislocation using “stepwise” in the R-package “RcmdrMisc”^19^ with BIC (Bayesian Information Criterion) as criterion for selection. To account for the variability of net UF of the last fill as well as the gcUF as a potential predictors for mechanical problems, we determined the expected values for both variables via linear regression for each patient for all seven days according to the method stated in a publication by Yang et al.^20^ on hemoglobin variability in 2007. Residuals of net UF in the last fill as well as the gcUF were calculated per patient day and summarized as standard deviation of the residuals (SD-Res) per patient. *P* values of < 0.05 were considered to be statistically significant.

## Supplementary Information

Supplementary Tables.
