# Publisher Correction: Assessing mechanical catheter dysfunction in automated tidal peritoneal dialysis using cycler software: a case control, proof-of-concept study

**DOI:** 10.1038/s41598-022-12074-y

**Published:** 2022-05-10

**Authors:** Krystell Oviedo Flores, Lukas Kaltenegger, Fabian Eibensteiner, Markus Unterwurzacher, Klaus Kratochwill, Christoph Aufricht, Franz König, Andreas Vychytil

**Affiliations:** 1 Division of Nephrology and Dialysis, Department of Medicine III, Medical University of Vienna, Währinger Gürtel 18-20, 1090 Vienna, Austria; 2 Baxter Healthcare GmbH, Vienna, Austria; 3 Division of Pediatric Nephrology and Gastroenterology, Department of Pediatrics and Adolescent Medicine, Comprehensive Center for Pediatrics, Medical University of Vienna, Vienna, Austria; 4 Christian Doppler Laboratory for Molecular Stress Research in Peritoneal Dialysis, Medical University of Vienna, Vienna, Austria; 5 Center for Medical Statistics, Informatics and Intelligent Systems, Medical University of Vienna, Vienna, Austria

Correction to: *Scientific Reports* 10.1038/s41598-022-09462-9, Published online 05 April 2022

The original version of this Article contained an error in the Results section under the subheading ‘Defining cut‑off values for prediction’ where,

“The parameters with the highest area under the curve (AUC) were number of total alarms (0.87, *P* = 0.0004), total drain time (0.82, *P* = 0.0017), and mean net UF of last fill (0.79, *P* = 0.0078.”

now reads:

“The parameters with the highest area under the curve (AUC) were number of total alarms (0.87, *P* = 0.0004), total drain time (0.82, *P* = 0.0017), and mean net UF of last fill (0.79, *P* = 0.0056).”

The original Article has been corrected.

