# Supplementary material for: Assessing mechanical catheter dysfunction in automated tidal peritoneal dialysis using cycler software: a case control, proof-of-concept study
Source: Sci Rep. 2022 Apr 5;12:5657. doi: 10.1038/s41598-022-09462-9 (PMC8983779; doi:10.1038/s41598-022-09462-9)
Supplement: Supplementary file 1 — Supplementary Tables. [file 41598_2022_9462_MOESM1_ESM.docx]

**Assessing mechanical catheter dysfunction in automated tidal peritoneal dialysis using cycler software: a case control, proof-of-concept study**

Krystell Oviedo Flores, MSc, MD; Lukas Kaltenegger, MD; Fabian Eibensteiner, MD; Markus Unterwurzacher, BSc; Klaus Kratochwill, PhD; Christoph Aufricht, MD; Franz König, PhD; Andreas Vychytil, MD

**Supplementary Table S1.** Clinical parameters assessed from the APD cycler card management software.

|  | Cases (*n*=14) | | Controls (*n*=19) | | |
| --- | --- | --- | --- | --- | --- |
| Parameter | **Mean (SD)** | **Median (IQR)** | **Mean (SD)** | **Median (IQR)** | ***P*-value** |
| Total alarms | 19.3 (16.5) | 13.5 (16.8) | 5.2 (4.0) | 5.0 (6.0) | 0.0002 |
| Total drain time (min) | 29.0 (11.3) | 24.3 (14.3) | 20.2 (6.8) | 17.7 (5.3) | 0.0012 |
| Net UF last fill ^a^ (mL) | -12.7 (153.3) | -10.0 (259.9) | 206.3 (228.4) | 227.6 (328.9) | 0.0047 |
| Days with negative UF last fill | 3.3 (2.6) | 3.0 (6.0) | 1.6 (2.1) | 1.0 (2.5) | 0.0749 |
| Net UF cycler ^b^ (mL) | 102.2 (372.2) | 160.5 (826.1) | 393.5 (445.8) | 242.1 (607.6) | 0.1320 |
| gcUF cycler ^b,c^ (mL/g/day) | 0.06 (2.3) | 0.42 (3.3) | 1.5 (2.9) | 1.0 (4.3) | 0.2258 |
| All patients performed tidal APD and received icodextrin-containing PD fluid for the daytime dwell, except for one patient in the control group who performed nightly intermittent PD without last fill.  ^a^ Corresponding to daytime dwell with icodextrin. ^b^ UF of last fill with icodextrin excluded. ^c^ Daily glucose corrected ultrafiltration (gcUF) was calculated as UF in mL divided by glucose load (total glucose in the fresh PD fluid infused each day) in grams. | | | | | |

**Supplementary Table S2.** Selected cut-off values for clinical parameters obtained from APD cycler card management software for predicting catheter dislocation in PD patients.

| Parameter | Cut-off value | Specificity (%) | Sensitivity (%) |
| --- | --- | --- | --- |
| Total alarms | > 7 | 85 | 79 |
| Total drain time (min) | >22 | 79 | 89 |
| Net UF last fil ^a^ (mL) | <150 | 93 | 66 |
| Days with negative UF last fill | >2 | 71 | 78 |
| ^a^ Corresponding to daytime dwell with icodextrin. | | | |
